# Progress and Perspective of CRISPR‐Cas9 Technology in Translational Medicine

**DOI:** 10.1002/advs.202300195

**Published:** 2023-06-25

**Authors:** Ruixuan Zheng, Lexiang Zhang, Rokshana Parvin, Lihuang Su, Junjie Chi, Keqing Shi, Fangfu Ye, Xiaoying Huang

**Affiliations:** ^1^ Joint Centre of Translational Medicine The First Affiliated Hospital of Wenzhou Medical University Wenzhou Zhejiang 325000 P. R. China; ^2^ Division of Pulmonary Medicine The First Affiliated Hospital Wenzhou Medical University Wenzhou Zhejiang 325000 P. R. China; ^3^ Wenzhou Key Laboratory of Interdiscipline and Translational Medicine The First Affiliated Hospital of Wenzhou Medical University Wenzhou Zhejiang 325000 P. R. China; ^4^ Oujiang Laboratory (Zhejiang Lab for Regenerative Medicine Vision and Brain Health); Wenzhou Institute University of Chinese Academy of Sciences Wenzhou Zhejiang 325000 P. R. China; ^5^ Beijing National Laboratory for Condensed Matter Physics Institute of Physics Chinese Academy of Sciences Beijing 100190 P. R. China

**Keywords:** clustered regularly interspaced short palindromic repeats‐associated protein 9 (CRISPR‐Cas9), gene regulation, gene therapy, translational medicine

## Abstract

Translational medicine aims to improve human health by exploring potential treatment methods developed during basic scientific research and applying them to the treatment of patients in clinical settings. The advanced perceptions of gene functions have remarkably revolutionized clinical treatment strategies for target agents. However, the progress in gene editing therapy has been hindered due to the severe off‐target effects and limited editing sites. Fortunately, the development in the clustered regularly interspaced short palindromic repeats associated protein 9 (CRISPR‐Cas9) system has renewed hope for gene therapy field. The CRISPR‐Cas9 system can fulfill various simple or complex purposes, including gene knockout, knock‐in, activation, interference, base editing, and sequence detection. Accordingly, the CRISPR‐Cas9 system is adaptable to translational medicine, which calls for the alteration of genomic sequences. This review aims to present the latest CRISPR‐Cas9 technology achievements and prospect to translational medicine advances. The principle and characterization of the CRISPR‐Cas9 system are firstly introduced. The authors then focus on recent pre‐clinical and clinical research directions, including the construction of disease models, disease‐related gene screening and regulation, and disease treatment and diagnosis for multiple refractory diseases. Finally, some clinical challenges including off‐target effects, in vivo vectors, and ethical problems, and future perspective are also discussed.

## Graphical abstract

Translational medicine is known as biomedicine models with purpose of finding solutions for clinical challenges. The clustered regularly interspaced short palindromic repeats associated protein 9 (CRISPR‐Cas9) system makes impressive strides therein through gene knockout, knock‐in, activation, interference, base editing, and sequence detection, and continues to revolutionize the development of translational medicine.

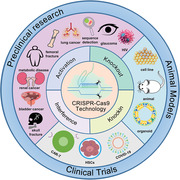

## 1 Introduction

Translational medicine is a biomedicine model, which emerged since 1990s with the purpose of finding solutions for clinical challenges using multidisciplinary approaches.^[^ ^1^, ^2^, ^3^ ^]^ Based on the previous research advances in genomics,^[^ ^4^ ^]^ artificial intelligence,^[^ ^5^ ^]^ immunology,^[^ ^6^ ^]^ epidemiology,^[^ ^7^ ^]^ oncology,^[^ ^8^ ^]^ and pharmacology,^[^ ^9^ ^]^ scientists and physicians have further unearthed the potential solutions for clinical challenges including determination of pathogenesis,^[^ ^10^ ^]^ diagnostic tools,^[^ ^11^, ^12^ ^]^ targeted agents,^[^ ^13^ ^]^ therapeutic strategies,^[^ ^14^, ^15^, ^16^, ^17^ ^]^ and systematic assessment of the feasibility, advantages, and disadvantages of novel applications.^[^ ^18^ ^]^ Currently, targeted agents based on mutated genes, for instance, gefitinib, dasatinib, and trastuzumab, have radically changed the treatment strategies for multiple tumor diseases and remarkably increased the survivals. However, editing therapies such as transcription activator‐like effector nucleases (TALENs) were not commonly deployed for clinical treatment yet.^[^ ^19^ ^]^ It is believed that severe off‐target effects, complex structure‐induced target site limitation and high research costs become the critical hinder factors.^[^ ^20^, ^21^ ^]^ Therefore, an efficient, accurate, convenient, and low‐cost gene editing tool is urgently anticipated to fulfill various research aims and to develop novel treatment techniques.

The clustered regularly interspaced short palindromic repeats‐associated protein 9 (CRISPR‐Cas9) system is coined as two parts: a guided sequence (CRISPR) and nuclease (Cas9).^[^ ^22^, ^23^ ^]^ In a particular CRISPR‐Cas9 system, CRISPR‐ribonucleic acid (crRNA) or single‐guide RNA (sgRNA) can precisely match target genes and cooperate with Cas9 nucleases for the cleavage reaction.^[^ ^24^ ^]^ Differed from other gene editing tools, the CRISPR‐Cas9 has the following advantages: a more straightforward structure, a broader editing site, lower cost, and a much lower off‐target efficiency owing to the principles of sgRNA and Cas9. Since the CRISPR‐Cas9 system was first implemented in mammalian cells in 2013,^[^ ^25^ ^]^ more than 20 000 articles have been published in PubMed. The capabilities of CRISPR‐Cas9 system covered gene knockout (KO),^[^ ^24^ ^]^ knock‐in (KI),^[^ ^26^ ^]^ activation,^[^ ^27^ ^]^ interference,^[^ ^28^ ^]^ screening,^[^ ^29^, ^30^ ^]^ specific sequence detection,^[^ ^31^ ^]^ and decontamination.^[^ ^32^ ^]^ The application ranges have expanded to pathogenesis and therapeutic gene screening for neoplastic diseases,^[^ ^33^, ^34^, ^35^, ^36^, ^37^, ^38^, ^39^, ^40^, ^41^ ^]^ infectious disease,^[^ ^42^, ^43^, ^44^ ^]^ hereditary diseases,^[^ ^45^ ^]^ and drug interactions, including anticancer drugs and immunomodulatory drugs.^[^ ^46^ ^]^

**Figure 1:**
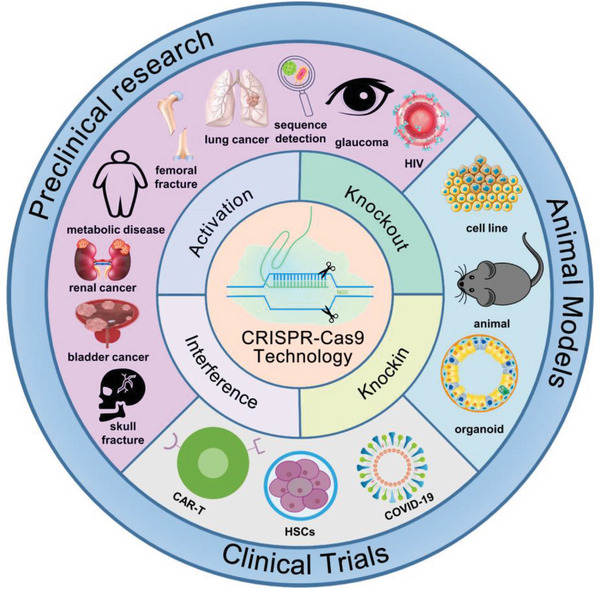
Overview of the significant achievements of the CRISPR‐Cas9 system in translational medicine.

In this review, we summarized the latest advances associated with CRISPR‐Cas9 in translational medicine. The first two sections introduce the mechanisms and highlights related to CRISPR‐Cas9 system. Subsequently, the third and fourth sections analyze potential preclinical research and clinical trials. Finally, the last two sections further consider the prospects and challenges of this gene editing therapy and provide constructive recommendations as for translational medicine deployment (**Figure** **1**).

## 2 CRISPR‐Cas9 and Other Gene Editing Tools

### 2.1 Dilemma of Zinc Finger Nucleases (ZFNs) and TALENs

ZFNs and TALENs are first‐generation gene‐editing tools that pioneered the era of gene editing, that comprise nuclease FokI and different DNA‐binding domains.^[^ ^47^ ^]^ The nucleases induce double‐strand breaks (DSBs) in DNA under the guidance of DNA‐binding domains. While ZFNs and TALENs have shown promise in preventing human immunodeficiency virus (HIV) infection,^[^ ^48^, ^49^ ^]^ their high off‐target rates have limited their broad application.^[^ ^50^, ^51^, ^52^ ^]^ Additionally, designing these tools is difficult and costly, with nonspecialists finding it challenging to engineer ZFNs due to the unspecified sequence affinity of zinc fingers and target site limitations associated with large amino acid units. Even open‐source ZFN components require at least six months of optimization time and a cost of $10 000.^[^ ^53^, ^54^, ^55^ ^]^ TALENs are cheaper and more precise due to their explicit sequence affinity, but their large size limits their use in complex processes. For example, their typical size's complementary DNA is ≈3 kb, and the load limit of the adeno‐associated virus (AAV) is only 4.7 kb, which impedes their use in simultaneous regulation of multiple genes. These limitations highlight the need for further research to develop more efficient and cost‐effective genome editing tools (**Table** **1**).^[^ ^54^, ^56^ ^]^

**Table 1:** Comparison of three generations gene editing tools

	ZFNs	TALENs	CRISPR‐Cas9
Nuclease	FokI	FokI	Cas9
Binding domains kind	Zinc finger	TALE repeat	crRNA (sgRNA)
Binding domain size	30s amino acids	30s amino acids	100 bp ssRNA
Recognition length	9 or 18 bp	18 bp	18–24 bp
Total size	1 kb	3 kb	4.2 kb
Delivery vector number	1	1	1 or 2
Identification specificity	low	Medium	High
Design difficulty	high	Medium	Low
Multiple gene regulation	unable	Unable	Able
Price for each gene	$15 000	$3000	$500

### 2.2 Principle and Characterization of CRISPR‐Cas9 System

The precise binding mechanism of sgRNA with the protospacer adjacent motif (PAM) sequence plays a important role in the accuracy of the CRISPR‐Cas9 working.^[^ ^57^, ^58^ ^]^ The sgRNA molecule consists of an 80‐nt RNA scaffold and a 20‐nt binding sequence that determines the cleavage location of the Cas9 nuclease in the genome.^[^ ^59^ ^]^ With the guidance of sgRNA, Cas9 nuclease cleaves the DNA strands three to four bases upstream of the PAM sequence, resulting in DSBs. The two major mechanisms that enable the fixation of the DSB and fulfill different purposes are nonhomologous end joining (NHEJ) and homology‐directed repair (HDR).^[^ ^60^ ^]^ NHEJ repair occurs in 95% of cells with DSBs, where cells “polish” the fracture and connect the nick. As base deletion induces a frameshift mutation, the protein loses its biological functions, enabling the knockout of the gene.^[^ ^61^ ^]^ By contrast, HDR is a more precise but time‐consuming repair method that requires a complete double‐stranded DNA donor template for homologous recombination. A donor template containing the target gene can be designed and delivered into DSBs to introduce the gene into a specific location, thereby achieving a gene knock‐in.^[^ ^62^, ^63^, ^64^ ^]^ Besides introducing or deleting gene segments, the CRISPR‐based cytosine base editors (CBEs) and adenosine base editors (ABEs) could also modify the gene sequence at single base pair resolution. The base editors consist of three critical components, i.e., deaminases, Cas9 nickase (nCas9), and sgRNA. Once combining to the targeted region, the cytosine (C) and adenosine (A) are deaminated to uracil (U) and inosine (I) by deaminases, and finally transform to thymine (T) and guanine (G) following complementary pairing and error correction processes.^[^ ^65^, ^66^ ^]^

The small size of sgRNA allows multiple sgRNAs to deliver with Cas9 nucleases in a vector and fulfill complex gene regulation purposes. Furthermore, the low design difficulty of the CRISPR‐Cas9 system allows for comprehensive application across various sites. The *S. pyogenes* Cas9 (SpCas9) system, which has the flexible PAM (NGG), only requires a 23‐nt sgRNA sequence to localize the target gene.^[^ ^67^ ^]^ Under optimized conditions, the editing efficiency could increase to 80% and the off‐target rates reduce to 0.05%.^[^ ^20^, ^57^, ^68^ ^]^ Nonetheless, in order to further expand the application scenarios of the CRISPR‐Cas9 system, high‐fidelity Cas9 variants, such as R691A high fidelity (HiFi) Cas9,^[^ ^69^ ^]^ and flexible Cas9 variants, such as *Streptococcus aureus* Cas9 (saCas9),^[^ ^70^ ^]^ SpCas9‐NG,^[^ ^71^ ^]^ and SpRY^[^ ^72^ ^]^ were discovered and fulfilled various requirements from different perspectives.

As a result of these advantages, numerous researchers rapidly transfer their attention to this novel tool and successfully improved treatment strategies in various fields.

## 3 Preclinical Research of CRISPR‐Cas9

**Figure 2:**
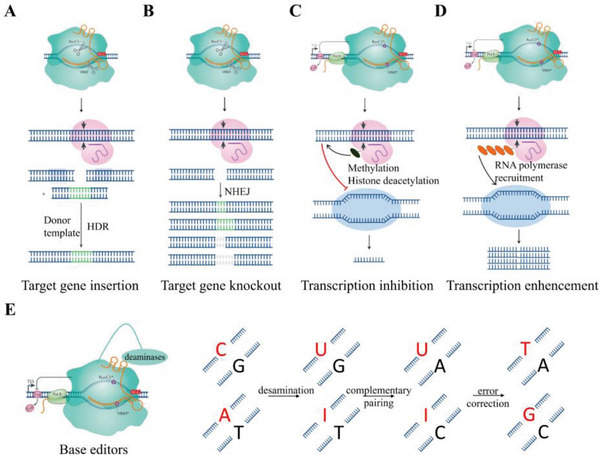
Mechanisms of different CRISPR‐Cas9 gene editing tools. Principal illustrations of A) gene knock‐in and B) knockout by different DSB repair methods. Schematic illustrations of C) gene knockdown, D) overexpression, and E) base editors by fusing different functional structure domains to nuclease‐dead Cas9 (dCas9).^[^ ^73^, ^74^ ^]^

By utilizing different repair mechanisms in DSB cells or fusing different functional domains to the dCas9 nuclease, we could regulate the biological behavior of cells from multiple dimensions, including gene knock‐in (**Figure** **2**A),^[^ ^75^ ^]^ knockout (Figure 2B),^[^ ^75^ ^]^ interference (Figure 2C),^[^ ^76^ ^]^ activation (Figure 2D),^[^ ^77^ ^]^ base editing (Figure 2E),^[^ ^78^ ^]^ and targeted delivery.^[^ ^79^ ^]^ Applications also across different diseases, such as neoplastic diseases,^[^ ^80^ ^]^ infectious diseases,^[^ ^81^ ^]^ hereditary diseases,^[^ ^82^ ^]^ and injurious diseases^[^ ^80^, ^83^ ^]^ have been reported.

### 3.1 Gene Knock‐In

Gene knock‐in is a rare event, typically less than 5% of cells with DSBs can integrate the desired gene through HDR, due to limitations associated with the synthesis phase (S phase) and the donor DNA deficiency.^[^ ^84^, ^85^ ^]^ However, recent advancements in CRISPR‐KI technology have enabled more controlled knock‐in processes, leading to successful applications in cell models,^[^ ^85^ ^]^ organoid models,^[^ ^86^ ^]^ and animal models.^[^ ^87^ ^]^ As the knock‐in process is becoming increasingly controlled, CRISPR‐KI technology has gradually been applied to monogenic genetic disorders with defined mutations such as cystic fibrosis (CF),^[^ ^88^ ^]^ familial hypercholesterolemia (FH),^[^ ^89^ ^]^ and sickle cell disease (SCD),^[^ ^90^ ^]^ and has demonstrated good results.

#### 3.1.1 Disease Models

Disease model is a critical tool to understand complex diseases and evaluate the efficacy of novel therapies.^[^ ^91^ ^]^ The CRISPR‐Cas9 system has facilitated the development of numerous disease models, including animal models,^[^ ^92^, ^93^ ^]^ organoid model,^[^ ^94^ ^]^ and cell models^[^ ^95^ ^]^ for neoplastic diseases, hereditary diseases, and so on. In the cyclization recombinase and locus of X‐over P1 (Cre‐*Lox*P) animal system, all of gene sequences among *Lox* were disrupted by Cre and lost their functions. However, the loss of gene segments specially exons also interfered the validation works of targeting therapy and gene editing therapy. Relying on double sgRNA CRISPR‐KI system, Huang et al. successfully replaced the mice alpha‐glucosidase (Gaa) gene as human infantile‐onset Pompe disease (IOPD) mutation sequence (c.1826dupA). Differing with rapidly progressive fatal cardiac in Cre‐*Lox*P IOPD mice model, the Gaa^c.1826dupA^ mice exhibited more human IOPB symptoms such as rapidly progressive fatal cardiac, and provide a suitable Gaa mutation site for the following research.^[^ ^93^ ^]^ Wall et al. introduced various mutant valosin‐containing protein (VCP) gene type into fly stocks to investigate the influence of VCP gene type in animal phenotypes. The various CRISPR‐based fly stock models revealed the distinct characteristics of VCP mutations, especially the influence in the function of mitochondria and endoplasmic reticulum.^[^ ^92^ ^]^ KRAS (Kirsten rat sarcoma viral oncogene homolog),^[^ ^95^ ^]^ p53 (tumor protein P53), and LKB1 (liver kinase B1)^[^ ^96^ ^]^ are the common mutated genes in lung adenocarcinoma. Relying on the lung‐targeted AAV serotypes, Platt et al. knocked out above‐mentioned genes and delivered a KRAS^G12D^ donor DNA to form a sporadic lung p53^−/−^, LKB1^−/−^, KRAS^−/−^, and KRAS^G12D^ mice model. After 2 months progression, multiple grades III bronchial alveolar adenomas were discovered and the sequencing results of tumor revealed the highest indel frequencies of p53 and LKB1, demonstrating the p53 and LKB1 were the main cause of this tumor occurrence rather than the KRAS^−/−^ and KRAS^G12D^.^[^ ^26^ ^]^ Furthermore, the CRISPR‐Cas9 system could also help to understand the interactions between genes and proteins. By inserting a fluorescent protein reporter into target genes, we can lead to better insights toward expression positions and interactions of specific genes.^[^ ^96^ ^]^ To investigate the biological activities of colon stem cells (CoSCs), a green fluorescent protein (GFP) lineage tracing cell line was constructed. By comparing the fluorescence distribution, they found that human CoSCs exhibited a lower regeneration rate than murine CoSCs.^[^ ^94^ ^]^ Differing with complex homologous recombination process in zygotes, the high convenience and accuracy of CRISPR‐based disease models seems more suitable to conduct high‐throughput screening.

#### 3.1.2 Disease Treatments

**Figure 3:**
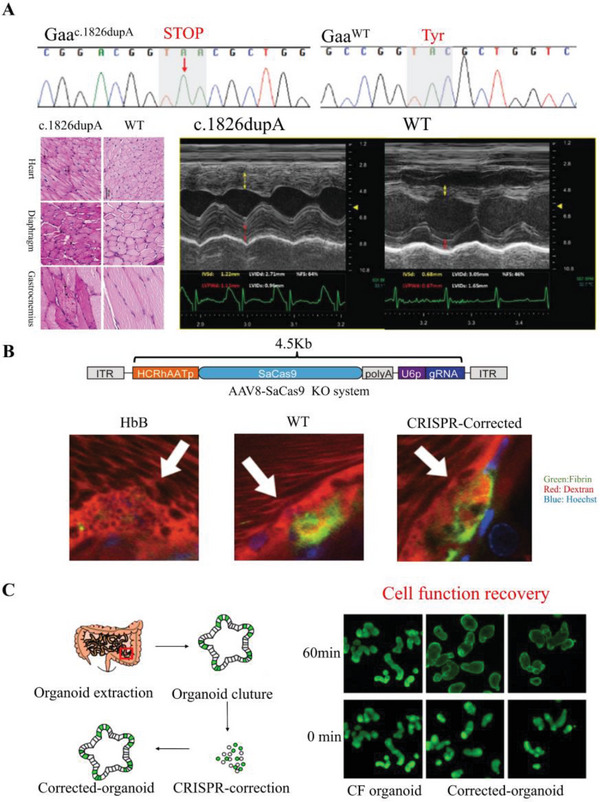
Schematic illustration and achievements of CRISPR‐Cas9 KI system in preclinical research. Experimental design and results of A) Gaa‐IOPD model generation. Reproduced with permission.^[^ ^93^ ^]^ Copyright 2020, Springer Nature. B) Exon2–8 knock‐in therapy for hemophilia B. Reproduced with permission.^[^ ^99^ ^]^ Copyright 2017, Springer Nature. C) CFTR knock‐in therapy for CF. Reproduced with permission.^[^ ^88^ ^]^ Copyright 2013, Elsevier Ltd.

CRISPR‐KI can also be used to introduce normal genes into mutant organisms, thereby restoring the normal function of the target genes. For instance, SCD is a single gene mutation induced anemia disease, where the Glu6Val mutation results in an abnormal appearance of hemoglobin B (HbB) rather than hemoglobin A (HbA).^[^ ^97^ ^]^ Autografting patients with CRISPR‐corrected hematopoietic stem cells (HSCs) have been shown to increase the number of corrected HSCs by fivefold 16 weeks after planting, with 45% of red blood cells having at least one HbA allele. These data indicate that corrected HSCs can work rapidly in transplant recipients and this function is maintained until the end of the experiment, providing a feasible option for patients with severe SCD.^[^ ^90^ ^]^ Similarly, CRISPR‐KI can be used to correct cystic fibrosis transmembrane conductor receptor (CFTR) mutations that cause CF. Introduction of wild‐type CFTR genes resulted in corrected organoids resuming the standard swelling rate of healthy CFTR and regaining sensitivity to channel inhibitors (**Figure** **3**D).^[^ ^88^ ^]^ In another study, the CRISPR‐KI system was used to correct the low‐density lipoprotein receptors (LDLR) E208X mutation in mice, which simulated the abnormal function of LDLR in FH.^[^ ^98^ ^]^ The CRISPR‐corrected LDLR significantly alleviated the hypercholesterolemia status of animal models, as evidenced by lower LDL‐cholesterol level and smaller atherosclerotic plaques volume (Figure 3B).^[^ ^89^ ^]^ Hemophilia B is a hemorrhagic disease caused by coagulation factor IX (F9) gene mutation. A complete exon 2–8 sequence was inserted into the intron1 region of F9‐deficient mice models. After knock‐in, the inserted genes significantly reversed the decrease in F9 and improved the bleeding time (Figure 3C).^[^ ^99^ ^]^ These results demonstrated that using CRISPR‐KI to correct mutated genes is a viable therapeutic strategy for inherited diseases and monogenic genetic disorders. However, these correction therapies require long‐term verification for potential side effects and off‐target correction to verify their feasibility and safety.

### 3.2 Gene Knockout

CRISPR‐KO systems offered a convenient for precise knockout of specific genes by NHEJ repair‐induced frameshift mutations. This technology has been extensively employed to investigate the functions of genes in various neoplastic diseases,^[^ ^80^ ^]^ infectious diseases,^[^ ^43^ ^]^ hereditary diseases,^[^ ^100^ ^]^ and verify the novel therapies based on previous conclusions.

#### 3.2.1 Neoplastic Diseases

Cancer is the deadliest disease due to its insidious initiation, rapid development, and poor prognosis. However, even the biological manifestations of the same tumor type may be completely different, proving that tumor behaviors are simultaneously regulated by multiple genes. Thus, various regulatory methods have been applied for various genes, such as oncogenes,^[^ ^34^ ^]^ drug resistance genes,^[^ ^35^, ^36^, ^37^, ^101^ ^]^ and metastasis‐related genes,^[^ ^102^ ^]^ in order to pave the way for the development of tumor therapy.

##### Oncogenes and Tumor Suppressor Genes

**Figure 4:**
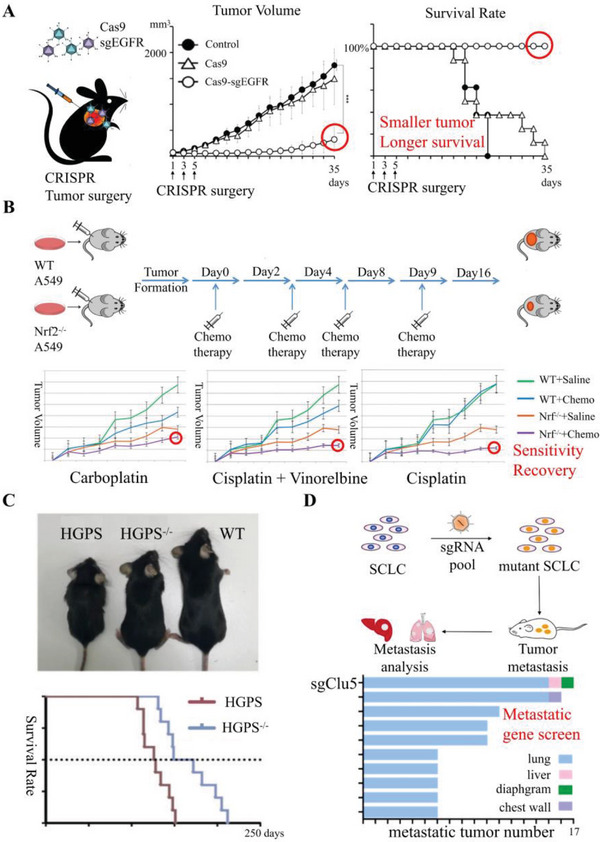
Schematic illustration and achievements of CRISPR‐Cas9 KO system in preclinical research. Experimental design and results of A) oncogene surgery therapy. Reproduced with permission.^[^ ^34^ ^]^ Copyright 2017, Oxford University Press. B) Drug sensitivity restoration therapy. Reproduced with permission.^[^ ^36^ ^]^ Copyright 2018, Elsevier Ltd. C) HGPS therapy. Reproduced with permission.^[^ ^16^ ^]^ Copyright 2019, Springer Nature. D) CRISPR‐screening based metastasis gene screening. Reproduced with permission.^[^ ^102^ ^]^ Copyright 2019, American Society for Clinical Investigation.

Abnormal expression of oncogenes and tumor suppressor genes is a hallmark of tumorigenesis. The deleterious effects of oncogene overexpression and tumor suppressor gene inhibition on tumor behavior have been extensively demonstrated. Thus, targeting oncogenes and tumor suppressor genes is a promising approach for cancer therapy. Koo et al. successfully deleted a panel of oncogenes in lung cancers, including those encoding for epidermal growth factor receptor (*EGFR*) (**Figure** **4**A), *CD38*, focal adhesion kinase (*FAK*), *NESTIN*, remodeling and spacing factor 1 (*RSF1*), and catenin delta 2 (*CTNND2*). The deletion of these oncogenes significantly reduced tumor volume and prolonged survival time in various nonsmall cell lung cancer (NSCLC) cell lines, providing a solid theoretical basis for the application of CRISPR‐based oncogene surgery therapy.^[^ ^34^ ^]^ Additionally, the high heterogeneity of tumors poses a significant challenge for targeted therapy. To address this issue, researchers have used CRISPR to synchronously knock out five tumor suppressor genes associated with acute myelogenous leukemia in HSCs, resulting in a model that accurately recapitulates tumor progression, the tumor microenvironment, and drug resistance reactions in leukemia patients. This multiple KO cell model represents a valuable tool for developing effective therapeutic strategies for leukemia.^[^ ^103^, ^104^ ^]^

##### Drug‐Resistant Genes

Drug resistance is a challenging issue in cancer therapy, and it is imperative to develop effective strategies to overcome it. To solve this issue, Heyza et al. and Yu et al. utilized CRISPR‐Cas9 technology to knock out genes encoding for *RSF1*, excision repair cross‐complementing 1 (*ERCC1*), nuclear factor erythroid 2‐related factor 2 (*NRF2*), and Aurora B in drug‐resistant NSCLC cell lines. Above study revealed that the drug‐resistant cells regained sensitivity to chemotherapeutic drugs, including paclitaxel, cisplatin, and carboplatin. This finding suggests that targeting these genes could potentially restore the efficacy of chemotherapy in drug‐resistant NSCLC (Figure 4B).^[^ ^35^, ^36^, ^37^, ^101^, ^105^ ^]^ Furthermore, CRISPR‐screening technology was utilized to identify phosphoglycerate dehydrogenase (PHGDH) as a suspected drug‐resistance gene against sorafenib for hepatocellular carcinoma. Inactivating PHGDH prolonged the progression‐free period of sorafenib, as well as regorafenib and lenvatinib.^[^ ^106^ ^]^ These results demonstrate the potential of CRISPR‐Cas9 technology for identifying drug‐resistance genes and developing new treatment strategies, such as combination therapy with sensitizers and chemotherapy drugs, or avoiding specific antineoplastic drugs for drug‐resistant tumors. The combination of CRISPR screening and transcriptome sequencing facilitates the filtration of target genes from the massive genome, providing a valuable tool for developing effective therapeutic strategies for cancer.

##### Metastasis Genes

Metastasis genes play a crucial role in determining the metastatic ability and propensity of tumors.^[^ ^107^ ^]^ Owing to the characteristics of “early metastasis”, there are no effective therapies for small cell lung cancer (SCLC). However, recent advances in CRISPR‐screening technology have identified the deletion of the gene encoding cullin5 (Cul5) as a potential target for reducing the formation of the Cul5‐suppressor of cytokine signaling (SOCS3) complex and increasing integrin *β*1 protein levels, which can promote SCLC metastasis. Importantly, dasatinib has been found to inhibit the metastatic activity of SCLC with Cul5 mutation, making it the first available targeted agent for patients with Cul5‐mutated SCLC. This provides a viable approach for subsequent screening of targeted agents for specific mutation types of tumors (Figure 4C).^[^ ^102^ ^]^ Fatty acid‐binding protein 4 (FABP4) is another critical regulator of lipometabolism that is involved in the intercommunication between tissues and lipid‐enriched regions.^[^ ^108^ ^]^ Studies have shown that the loss of FABP4 can significantly inhibit the lipid‐enriched metastasis tendency of ovarian cancer cells, resulting in a significant reduction in metastases in the abdominal adipose region.^[^ ^33^ ^]^ For highly malignant tumors, such as SCLC and undifferentiated thyroid cancer, unobserved early distant metastases are the most crucial factors leading to an uncontrollable state. However, the inhibition of metastatic genes can be incorporated into treatment strategies, which may considerably delay the metastasis of highly malignant tumors and provide patients with a long survival time or a possible cure.

#### 3.2.2 Infectious Diseases

Persistent and latent infections associated with chronic infectious diseases pose significant challenges to achieving complete cure.^[^ ^109^ ^]^ In patients with acquired immune deficiency syndrome (AIDS), the virus prefers to infect and integrate self‐gene fragments into host cells with specific receptors.^[^ ^110^ ^]^ Hence, several researchers have knocked out the aforementioned receptor genes in induced pluripotent stem cells,^[^ ^42^ ^]^ TZM‐Bl,^[^ ^43^ ^]^ hematopoietic stem cells (HSPCs),^[^ ^43^ ^]^ and primary CD4(+) T cells to reduce the infection rate of HIV (Figure 4D). After conversion, the immune functions of KO cells remained normal, but the infection resistance against HIV was significantly improved, demonstrating the great potential of completely cure for AIDS.^[^ ^43^, ^44^ ^]^ A critical challenge in hepatitis B virus (HBV) infection is the difficulty in eliminating HBV‐DNA using antiviral drugs. Stone et al. built an AAV system to target the HBV genome and reduce the total number of HBV. In successfully edited mice, the liver function and hepatocyte survival rates were significantly improved, and the DNA and covalently closed circular DNA of HBV also showed a downward trend.^[^ ^111^ ^]^ The CRISPR‐Cas9 system provides a novel solution for diseases that lack sensitive drugs and organs that are challenging to treat with drugs. Despite challenges such as immune system interference and the complex human body environment, we remain optimistic about the future of these studies.

#### 3.2.3 Hereditary Diseases

Hereditary diseases are often caused by gene mutation induced protein functional alteration. In the case of SCD, Liu et al. proposed a novel hypothesis to address this issue. They knocked out the B‐cell lymphoma/leukemia 11A (BCL11A) gene of HSPCs, which is a type of hemoglobin F (HbF) repressor. The deficiency of BCL11A did not affect the normal function of HSPCs but promoted HSPCs differentiation to HbF, which can work with the HbB mutation. These findings reveal how BCL11A initiates competitive repression and provides potential therapeutic targets by inhibiting the HbB differentiation.^[^ ^100^ ^]^ Another example is the abnormal gene sequence of lamin A/C (LMNA), which often induces the occurrence of Hutchinson‐Gilford progeria syndrome (HGPS). Santiago‐Fernández et al. proposed that knocking out LMNA exon 11 could extend the median survival duration (from 127 to 160.5 days), along with the reduction of premature aging in the LMNA^G609G/G609G^ mouse model (Figure 4E).^[^ ^16^ ^]^ These findings are inspiring as they demonstrate the critical role of accessibility and high specificity in understanding the biological functions of genes and implementing effective protocols that further promote the development of in vivo editing therapy and targeted therapy.

### 3.3 Gene Interference

**Figure 5:**
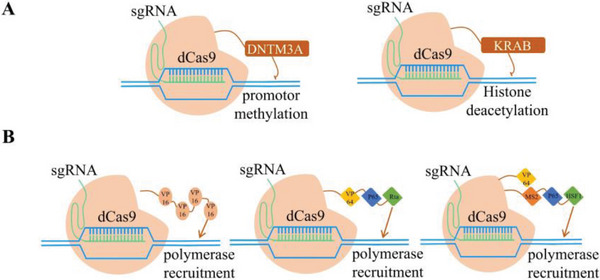
Mechanisms of different dCas9 transcription regulation tools. Principal illustrations of A) dCas9‐DNTM3A and dCas9‐KRAB transcription interference tools and B) dCas9‐VP64, dCas9‐VPR, and dCas9‐SAM transcription activation tools.

The dCas9 nuclease is a mutated Cas9 protein that loses nuclease activity but can still bind to sgRNA and exert a targeting effect. Based on this, different transcription inhibition functional domains, such as methylation proteins and histone deacetylation proteins, have been fused to regulate target genes, which is known as CRISPR interference (CRISPRi) (**Figure** **5**A).^[^ ^112^ ^]^

#### 3.3.1 RNA Interference (RNAi) and CRISPRi

RNAi is a commonly used tool for suppressing gene expression, but it has limitations such as incomplete, transient, and low specificity, and always only one gene can be regulated in a single cell. Therefore, RNAi application is significantly limited.^[^ ^73^, ^113^ ^]^ However, the emergence of CRISPRi has reversed this situation. In CRISPRi systems, multiple sgRNAs can be used to regulate one gene or different genes simultaneously, resulting in enhanced inhibitory effects and complex purposes. These characteristics provide researchers multiple options to fulfill diverse purposes.^[^ ^28^, ^112^, ^114^, ^115^ ^]^

#### 3.3.2 Methylation Inhibition

DNA methyltransferase 3A (DNMT3A)‐induced promoter methylation is a well‐established epigenetic pathway in humans.^[^ ^116^ ^]^ Vojta et al. successfully applied dCas9‐DNMT3A in HEK293 cells to inhibit mRNA transcription and protein expression of two genes, demonstrating the effectiveness of dCas9‐DNMT3A as a gene‐regulating tool that represses target genes while maintaining genomic integrity.^[^ ^117^, ^118^ ^]^ Desmoplakin (DSP) has been identified as a potential risk factor for idiopathic pulmonary fibrosis due to high levels of expression and diminished methylation. To address this issue, Qu et al. utilized dCas9‐DNMT3A to reestablish the methylation system of DSP, resulting in the reversal of abnormal expression and better pulmonary morphological manifestations.^[^ ^119^ ^]^ Additional studies also demonstrated the potential of dCas9‐DNMT3A in inhibiting tumor‐related behavior by downregulating human telomerase reverse transcriptase and locomotor proteins of SWIA/SNF‐related, matrix‐associated, actin‐dependent regulator of chromatin, subfamily a, member 2 (SMARCA2) in melanoma cells and lung adenocarcinoma.^[^ ^120^, ^121^ ^]^ These findings highlight the versatility of dCas9‐DNMT3A as an inhibitory tool in various cell types and suggest its potential use in exploring disease pathogenesis and identifying potential drug targets to improve patient outcomes.

#### 3.3.3 Krüppel‐Associated Box (KRAB) Inhibition

**Figure 6:**
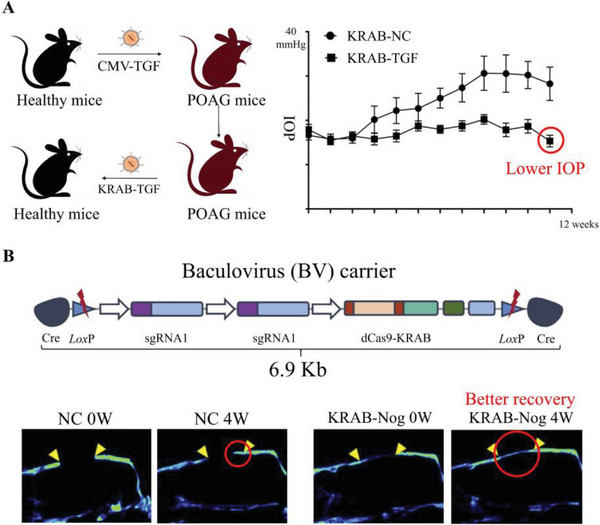
Schematic illustration and achievements of CRISPRi system in preclinical research. Experimental design and results of A) TGF‐*β*2 knockdown therapy for POAG. Reproduced with permission.^[^ ^125^ ^]^ Copyright 2021, Association for Research in Vision and Ophthalmology. B) Noggin knockdown therapy for bone fracture. Reproduced with permission.^[^ ^126^ ^]^ Copyright 2021, Elsevier Ltd.

Histone deacetylation is an important epigenetic mechanism that inhibits transcription factor binding by increasing the ribosomal helix degree. The KRAB repressors are functional proteins involved in the histone deacetylation.^[^ ^122^, ^123^ ^]^ The unexplained hyperacetylation status of transforming growth factor beta‐2 (TGF‐*β*2) is one of the important cause of primary open‐angle glaucoma (POAG).^[^ ^124^ ^]^ Rayana et al. successfully suppressed TGF‐*β*2 by deacetylating the promoter region of the TGF‐*β*2 gene, which reversed the occurrence of ocular hypertension and glaucoma. It is important to note that partial inhibition of TGF‐*β*2 is a safer and more effective treatment strategy for patients with POAG than complete knockout, as the normal level of TGF‐*β*2 is necessary to maintain ocular homeostasis (**Figure** **6**A).^[^ ^125^ ^]^ Large calvarial bone defects are a challenge for both young and old patients. Inhibition of Noggin expression in adipose‐derived stem cells eliminated the negative feedback inhibition of bone morphogenetic protein 2 (BMP2), resulting in improved fracture healing rates by promoting BMP2‐induced new bone mineralization and osteogenic differentiation (Figure 6B).^[^ ^126^ ^]^ Apart from mRNA, the expression levels of microRNAs (miRNAs) can also be regulated by the dCas9‐KRAB.^[^ ^127^ ^]^ The binding of dCas9‐KARB to the pri‐miR3662 region significantly inhibited the level of miR‐3662 and suppressed the progression in triple‐negative breast cancers (TNBC) by regulating the HMG‐box transcription factor 1 (HBP1)/N‐*β*‐catenin/c‐Myc axis.^[^ ^128^ ^]^ Furthermore, recent studies also reported the advances for the dCas13‐based RNA methylation regulators, including dCas13‐M3 and dCas13‐M3M14, which enabled to change transcript abundance and further regulate the post‐translational modification process.^[^ ^129^ ^]^ CRISPRi has demonstrated its effectiveness in regulating methylation and histone deacetylation to modulate protein and microRNA (miRNA) expression. Compared to complete gene knockout by CRISPR‐KO, CRISPRi offers a distinct advantage in that the suppressed protein can still be expressed at low levels, which is crucial for genes necessary for survival. Moreover, the degree of inhibition of CRISPRi also can be controlled by adjusting different functional domains and target regions, allowing for high‐precision regulation.

### 3.4 Gene Activation

CRISPR activation (CRISPRa) is mediated by dCas9 with different transcriptional activation functional domains, such as VP64, synergistic activation mediator (SAM), SunTag, and VPR (Figure 5B).^[^ ^130^, ^131^ ^]^ When dCas9 binds to a target gene, the recruited RNA polymerase increases the expression of the target gene in a transcription‐promoting manner.^[^ ^132^, ^133^ ^]^

#### 3.4.1 VP64

**Figure 7:**
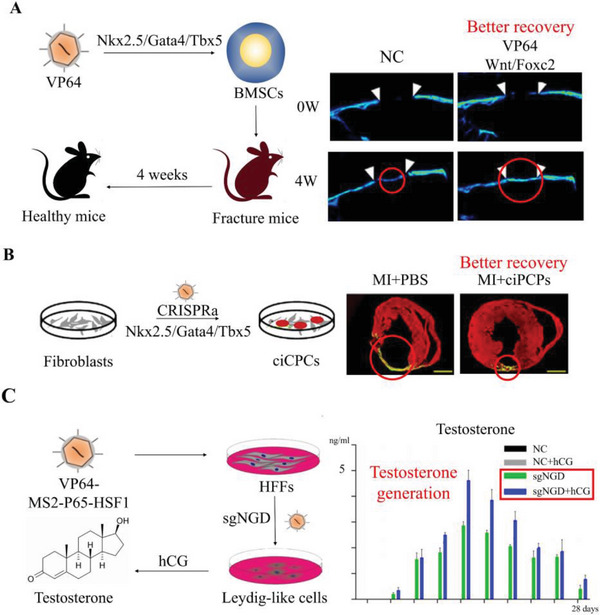
Schematic illustration and advancements associated with CRISPRa system in preclinical research. Experimental design and results of A) Wnt/Foxc2 coactivation therapy for bone fracture. Reproduced with permission.^[^ ^141^ ^]^ Copyright 2020, Elsevier Ltd. B) Nkx2.5, Gata2, and Tbx5 coactivation therapy for MI. Reproduced with permission.^[^ ^142^ ^]^ Copyright 2022, Elsevier Ltd. C) NGD coactivation therapy for male hypogonadism. Reproduced with permission.^[^ ^137^ ^]^ Copyright 2019, John Wiley & Sons, Inc.

dCas9‐VP64 is the first generation and the most widely used CRISPRa because of its modest activation effect.^[^ ^132^ ^]^ Saayman et al. demonstrated the potential of CRISPRa in HIV functional cure strategy by activating the long terminal repeat to maintain the constant activation state of HIV.^[^ ^134^, ^135^ ^]^ Leydig cell (LC) transplantation is a promising therapy for male hypogonadism. However, lack of “seed cells” seriously impeded the development of transplantation therapy.^[^ ^136^ ^]^ The dCas9‐VP64 system activates the nuclear receptor subfamily 5 group A member 1 (Nr5a1), GATA binding protein‐4 (Gata4), and double sex, and mab‐3 related transcription factor 1 (Dmrt1) to reprogram human foreskin fibroblasts (HFFs) as LC‐like cells. After reprogram process, 10% of HFFs were converted into LC‐like cells and rapidly capable for androgen secretion, providing novel evidence for male hypogonadism endogenous therapy (**Figure** **7**C).^[^ ^137^ ^]^ Moreover, to improve the irreversible decline in cardiac function after myocardial infarction (MI), a range of cardio‐associated differentiation factors were activated in cardiosphere‐derived cells (CDCs) using dCas9‐VP64. This resulted in the generation of activated CDCs that exhibited cardiomyocyte characteristics and significantly improved ejection fraction and myocardial ischemia in patients with MI (Figure 7B).^[^ ^138^ ^]^

#### 3.4.2 Synergistic Activation Mediator

dCas9‐SAM is a transcriptional activation complex that fuses with various transcriptional activators, including VP64, MS2, p65, and heat shock transcription factor 1.^[^ ^139^ ^]^ Limsirichai et al. reactivated HIV using dCas9‐SAM and obtained similar results to those of Saayman et al.^[^ ^140^ ^]^ Severe fractures are a major challenge for the elderly, the simultaneous activation of Wnt family member 10b (Wnt10b) and Forkhead Box 2 (Foxc2) using dCas9‐SAM remarkably enhanced the bone healing capacity of bone marrow‐derived mesenchymal stem cells (BMSCs) in critical‐sized calvarial defect rats (Figure 7A).^[^ ^141^ ^]^ Another study reported that dCas9‐SAM reprogramed fibroblasts into cardiovascular progenitor cells (CPCs) by activating Gata4, Nkx2‐5, and T‐Box transcription factor‐5 (Tbx5) and enabled reprogramed CPCs to differentiate into cardiovascular cells in the infarcted heart to improve heart ischemia and heart function.^[^ ^142^ ^]^ The complex pathogenic mechanism of islet stem cells presents a significant obstacle to regenerative therapy for patients with diabetes mellitus. To address this, researchers applied the dCas9‐SAM to activate the uncoupling protein 1 gene and induce the conversion of white adipocytes into brown‐like adipocytes. This improved glucose homeostasis, significantly controlled the increased rate of blood glucose and body weight under a high‐calorie diet, and led to adipose tissue H&E staining and immunostaining that showed brown‐like adipocyte features.^[^ ^143^ ^]^

#### 3.4.3 VPR

VPR, which stands for VP64‐P65 and Rta, refers to complexes formed by three types of transcriptional activators.^[^ ^144^ ^]^ In a study by Böhm et al., dCas9‐VPR was used to activate the opsin 1, medium wave sensitive (Opn1mw) gene to compensate for rhodopsin defects and treat inherited retinal dystrophies (IRDs). The activation of Opn1mw significantly improved the retinal degeneration rate and retinal function in an IRD mouse model, with no apparent adverse reactions observed during the one‐year treatment period.^[^ ^145^ ^]^ The dCas9‐based activation system has a unique capacity to regulate multiple genes simultaneously by loading multiple sgRNAs in one vector, paving the way for fulfilling more complex tasks such as changing the differentiation tendency of cells, enhancing treatment effects, and dissecting complex pathological mechanisms. Unlike traditional activation systems such as plasmids or virus, the size limitation and infection efficiency make multiregulation in a single cell uncontrollable. Thus, the above results highlight the potential of dCas9‐based activation systems in therapeutic applications for various diseases (**Table** **2**).

**Table 2:** Typical preclinical applications of CRISPRa and CRISPRi in recent three years

Year	Disease	Target cell	Target gene	Functional domain	Vector and model	Major findings	Refs.
2022	Alzheimer's disease	MEF, NIH/3T3	APP	DNMT3A	Lentivirus cell and animal	Methylation of APP can effectively reverse the symptoms of Alzheimer's disease.	[146]
2019	Lung cancer	H1299	SMARCA2	DNMT3A	Lentivirus cell model	SMARCA2 is a tumor suppressor gene, and hypermethylation can drive lung cancer development.	[121]
2021	Ocular hypertension	TM	TGF*β*2	KRAB	Lentivirus cell and animal	dCas9‐KRAB system can effectively reverse the TGF*β*2‐induced OHT.	[125]
2021	Bladder cancer	SVHUC‐1, T24, UMUC3, BIU‐87	CacyBP	KRAB	Plasmid cell and animal	*CacyBP* is an oncogene associated with bladder cancer, and the inhibition of *CacyBP* by the dCas9‐KRAB system can be a therapeutic approach.	
2022	Breast cancer	TNBC	miR‐3662	KRAB	Lentivirus cell and animal	dCas9‐KRAB system can be a therapeutic approach to treat miR‐3662 dysregulation in patients with TNBC.	[128]
2021	TDT SCD	HUDEP‐2	BCL11A	VP64	Lentivirus cell model	The dCas9‐VP64 system can activate BCL11A to promote the switch from fetal to adult globin.	[100]
2019	Obesity Diabetes	3T3‐L1	FABP4	VP64	Fusion peptide cell and animal	The dCas9‐VP64 system can activate FABP4 and reverse obesity‐induced metabolic syndromes.	[147]
2020	Bone fracture	BMSC	WNT10b, Foxc2	SAM	Baculovirus cell model	dCas9‐SAM system can activate Wnt10b and Foxc2 in BMSCs and remarkably improve calvarial bone healing.	[141]
2022	Myocardial infarction	Fibroblast	GATA4, NKX2‐5, TBX5	SAM	Lentivirus cell model	dCas9‐SAM system can activate multiplex genes at same time and start the reprogramming process of CPC.	[142]
2021	Cancer disease	HeLa, HEK293T	HBG1, FASLG, RAIL, SIM1, LATS2, DMD, MIAT	VPR	Picasso cell and animal	Picasso system shows a powerful capacity of loading huge size CRISPR‐Cas9 system.	[148]
2020	Inherited blindness	661 W, MEF	OPN1MW, CNGA1	VPR	Baculovirus cell and animal	The dCas9‐VPR system can be a therapeutic approach to treat *Opn1mw* (+) retinitis pigmentosa.	[145]

APP, amyloid precursor protein; HBG, hemoglobin subunit gamma; FASLG, Fas ligand; SIM, single‐minded family BHLH transcription factor; LATS2, large tumor suppressor kinase; DMD, dystrophin; MIAT, myocardial infarction‐associated transcript; CNGA1, cyclic nucleotide‐gated channel subunit alpha; ECS, embryonic stem cells; TM, trabecular meshwork; HUDEP, human umbilical cord blood‐derived erythroid progenitor; MEF, mouse embryonic fibroblasts.

### 3.5 Base Editors

Given the characteristics of ABEs and CBEs, one of the main applications of base editor therapy is base‐mutation‐induced hematological system diseases and neurological diseases.^[^ ^78^ ^]^ For instance, in SCD patients, the CAC base mutation is the root causes of HbB mutation. Newby et al. applied engineered ABEs to edit the CAC as CGC in SCD‐HSPCs, which generated another kind of nonpathogenic HbB called Makassar HbB (mHbB). Sixteen weeks after SCD mice receiving edited HSPCs, the blood cell lysates experiments revealed that mHbB presented 79% of total HbB and the hematologic parameters test also reflected that the red blood functions were almost improved (86%).^[^ ^149^ ^]^ Similarly, base editors have also been used to correct the point mutation of HGPS, where the progerin is caused by the C to T substitution (c.1824) in the LMNA gene. In vivo editing therapy using lentivirus ABE successfully corrected more than 90% of the mutant LMNA gene, leading to better blood elasticity, higher animal vitality, and longer survival.^[^ ^150^ ^]^ Base editing therapy offers distinct advantages over KI or KO therapy, including no DSB, no donor DNA, and lower influence on surrounding genes. However, the therapy is still in its infancy stage, and a systematic evaluation of treatment effects and potential side‐effects is crucial before its adoption as the first treatment choice for point mutation diseases.

### 3.6 Targeted Delivery of CRISPR‐Cas9

The delivery route remains a crucial aspect of its clinical application, and the search for a suitable delivery system of CRISPR‐Cas9 is ongoing. While plasmid and virus vectors have been utilized, their potential systemic effects, insertional mutagenesis, and immune reactions have limited their use. Moreover, their short blood cycle durations may further impact their efficacy. However, novel nonviral vectors have emerged as a promising solution to overcome these challenges.^[^ ^151^ ^]^ Polymer nanoparticles, liposomes, and lipid nanoparticles have complex structures that enable them to resist enzymatic degradation in the blood and target specific tissues or organs. These properties enhance the capacity of nonviral vectors to achieve more complex objectives.^[^ ^152^, ^153^, ^154^ ^]^

#### 3.6.1 Polymer Nanoparticles (NPs)

**Figure 8:**
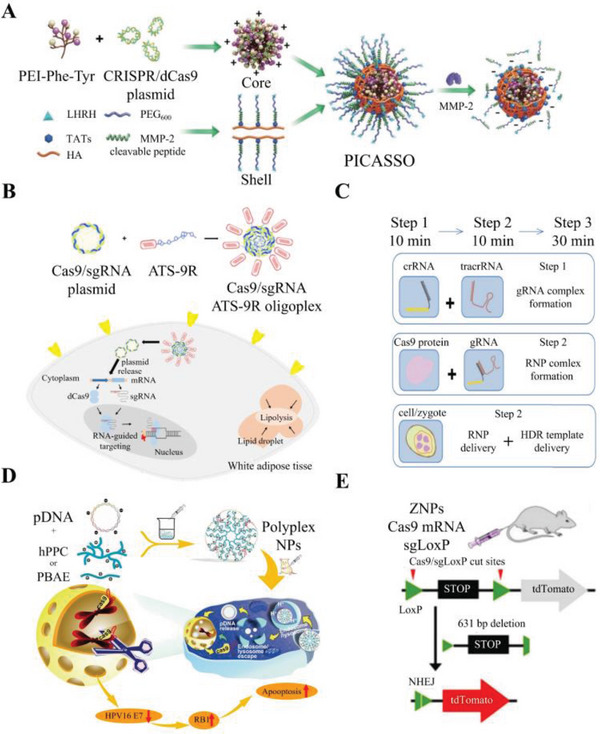
Schematic illustration of nonviral CRISPR‐Cas9 vectors. Schematic illustration of A) PICASSO. Reproduced with permission.^[^ ^148^ ^]^ Copyright 2021, Ivyspring International Publisher. B) ATS‐9R‐Cas9. Reproduced with permission.^[^ ^147^ ^]^ Copyright 2019, Cold Spring Harbor Laboratory Press. C) Lipofectamine vector. Reproduced with permission.^[^ ^159^ ^]^ Copyright 2017, Elsevier Ltd. D) PBAE‐Cas9. Reproduced with permission.^[^ ^156^ ^]^ Copyright 2020, Elsevier Ltd. E) ZNPs‐Cas9. Reproduced with permission.^[^ ^162^ ^]^ Copyright 2017, John Wiley & Sons, Inc.

Polymer NPs are colloidal particles with sizes ranging from 1 to 1000 nm. Their macromolecular structure enables them to carry larger DNA molecules and the lipid‐soluble external facilitates ability to cross cell membranes.^[^ ^155^ ^]^ Chung et al. have developed a novel fusion peptide that combines the adipocyte targeting sequence 9‐mer arginine (ATS‐9R) with a dCas9‐sgRNA plasmid. This innovative fusion peptide has shown promising results in mitigating several metabolic disorders, including obesity, insulin resistance, metabolic syndrome‐induced inflammation, and fatty liver disease (**Figure** **8**B).^[^ ^147^ ^]^ Gao et al. have improved the efficiency and reduced toxicity of Cas9‐sgRNA plasmid delivery through the use of polyplex nanoparticles with a poly(amide‐amine)–poly (*β*‐amino ester) hyperbranched copolymer outer shell, referred to as PBAE. Their study showed that PBAE‐Cas9 significantly reduced the growth rate of cervical cancer in both cell and animal models, suggesting its potential as a delivery vector for cervical cancer and human papillomavirus (HPV)‐related cancers (Figure 8D).^[^ ^156^ ^]^ Similarly, Liu et al. developed a programmable hierarchical‐responsive nanoCRISPR (PICASSO) nanoparticle with different core and shell parts. They demonstrated that the shell structure of PICASSO reduced blood circulation time‐induced degradation and improved tissue accumulation capacity, while the core structure increased the plasmids’ ability to prevent lysosomal enzyme action (Figure 8A).^[^ ^148^ ^]^ These studies, along with others utilizing different NP structures, demonstrate the potential of CRISPR‐NPs to overcome gene size restrictions, tissue targeting limitations, and lower hepatotoxicity compared to typical plasmids.

#### 3.6.2 Liposomes/Lipid Nanoparticles (LNPs)

In previous research, delivering mRNA into cells was a difficult issue because it was nuclease degradation effect and difficulty in cell membrane transporting.^[^ ^157^ ^]^ Several research teams have utilized lipofectamine, a common commercialized liposome carrier, to deliver Cas9‐sgRNA RNPs into difficult‐to‐transfect cells, such as somatic cells, stem cells, and zygotes (Figure 8C).^[^ ^158^, ^159^, ^160^ ^]^ However, lipofectamine has a strict size limitation and can only deliver short fragments. To overcome this limitation, different phospholipid structures and nanostructures have been used.^[^ ^161^ ^]^ A novel zwitterionic amino lipids (ZALs) system capable reduced the expression of target proteins by up to 90% and demonstrated successful introduction of the tdTomato gene into liver cells. The potent carrying capacity of ZALs allows for packaging of multiple long RNAs, making it a versatile platform for a wide range of applications (Figure 8E).^[^ ^162^ ^]^ The use of liposomes and LNPs with the Cas9‐sgRNA RNP delivery system has been shown to significantly increase efficiency and effectiveness, as they can enter the cytoplasm by high lipid solubility. Thus, liposomes and LNPs may be more feasible vectors for the Cas9‐sgRNA RNPs than virus vectors.

## 4 Clinical Applications of CRISPR‐Cas9

### 4.1 Clinical Therapies

After undergoing several years of preclinical studies, the CRISPR‐Cas9 therapy has finally entered the clinical trial phase. In comparison to other gene editing tools such as ZFN, TALENs, AAV, and RNAi, the CRISPR‐Cas9 system exhibits higher specificity, accessibility, and controllability, making it a promising candidate for gene therapy. The first successful clinical trial involving the delivery of autogenous programmed cell death‐1 (PD‐1)‐KO therapy to treat NSCLC patients was reported by Lu et al.^[^ ^38^ ^]^ Since then, over ten clinical trials are currently underway, and initial results have been promising.

#### 4.1.1 NSCLC

PD‐1 is a critical immune checkpoint receptor that inhibits immune cell activity by regulating T‐cell exhaustion.^[^ ^163^, ^164^ ^]^ Lu et al. administrated the autogenous PD‐1‐KO T cell into patients and observed the survival‐related parameters including median progression‐free survival (PFS) and overall survival (OS). The average waiting time for the knockout process was 25 days with a 77.3% culture success rate. The PFS and OS were 7.7 and 42.6 weeks, respectively, which were significantly higher than conventional therapy. The off‐target rate is as low as 0.05%, with only grade 1 or grade 2 adverse events.^[^ ^38^ ^]^ This was the first time when CRISPR‐Cas9 therapy was applied to humans. The results demonstrated that clinical CRISPR‐Cas9 therapy is feasible for improving treatment efficacy.

#### 4.1.2 Relapsed/Refractory Acute Lymphoblastic Leukemia (r/r ALL)

The emergence of chimeric antigen receptor T (CAR‐T) therapy has rapidly changed difficult situations of ALL patients.^[^ ^165^, ^166^ ^]^ Nevertheless, a significant proportion of patients still experience disease relapse within one year of receiving CAR‐T therapy due to tumor resistance and poor persistence of CAR‐T cells.^[^ ^167^, ^168^ ^]^ Stadtmauer et al. utilized a CRISPR‐Cas9 system to knock out PD‐1 endogenous T‐cell receptor (TCR) chains and link a cancer‐related TCR chain to enhance the killing capacity of edited T cells. Patients achieved the longest stabilization time of more than 100 days and a 50% reduction in large abdominal masses. The edited T cells persisted for up to nine months without cytokine release syndrome in three patients, and the off‐target rate also gradually decreased over three months.^[^ ^39^ ^]^ Additionally, a universal CD19/CD22 dual targeting CAR‐T product was developed using a CRISPR‐Cas9 system to overcome the challenges associated with weak primary cell activity, long waiting times for cell cultures, and high cost. The complete remission rate was over 80% and with no prominent adverse events. However, all patients experienced cytokine release syndrome.^[^ ^169^ ^]^ These findings provide a promising avenue for the development of effective treatments for patients with relapse and progression stage ALL.

#### 4.1.3 Transthyretin Amyloidosis (ATTR)

Transthyretin amyloidosis is a monogenic hereditary disease resulting from the accumulation of abnormal transthyretin protein, leading to organ dysfunction and death.^[^ ^170^ ^]^ As the liver is the primary site for transthyretin (TTR), a hepatocyte‐targeted LNP was developed to deliver Cas9‐sgRNA mRNA precisely into hepatocytes and alleviate TTR retention. In a clinical trial involving 172 patients, the TTR level decreased from day 14 and reached its lowest level at day 28. The remission effect of the symptoms was dose‐dependent, and even the low‐dose group showed a remission effect of more than 50%. These results provide a promising therapeutic approach for patients with transthyretin amyloidosis.^[^ ^171^ ^]^

#### 4.1.4 SCD and TDT

Frangoul et al. conducted a study where they edited CD34+ HSPCs by knocking out BCL11A and then transplanted the edited HSPCs into patients with transfusion‐dependent TDT and SCD under myeloablative conditions. Following the transplantation of the edited HSPCs, HbF levels in patients increased rapidly to 95% in the fourth month and remained at 99% until the 18th month. The sequencing results from the 18th month showed that all mutation sites were consistent with expectations, indicating that off‐target editing was maintained at a reasonably low level.^[^ ^45^ ^]^ GATA1, an enhancer of BCL11A gene, was also knocked out in HSPCs, resulting in the reversal of *β*0/*β*0 TDT patients. After an 18‐month follow‐up, two patients showed an increase in HbF levels to 15.0 and 14.0 g dl^−1^, with over 85% editing persistence in bone marrow cells. These findings present a promising therapeutic approach and safety for patients with TDT and SCD (**Table** **3**).^[^ ^172^ ^]^

**Table 3:** Summary of recent CRISPR‐Cas9 based gene editing therapy clinical trials

NCT number as reference	Disease	Gene	Intervention	Participants number	Phase	Trial target	Status
NCT04560790	Refractory viral keratitis	VEGFA	VLP‐mRNA (BD111)	6	1/2	To verify the clinical feasibility of VLP‐mRNA vector for refractory viral keratitis	Active, not recruiting
NCT04990557	COVID‐19	PD‐1 ACE2	Engineered T cells	16	1/2	To verify the clinical feasibility of PD‐1 and ACE2 KO T cell for long‐term immunity of COVID‐19	Not yet recruiting
NCT03057912	Cervical intraepithelial neoplasiaI	E6 E7	plasmid	60	1	To verify the clinical feasibility of HPV E6/E7‐KO therapy for cervical intraepithelial neoplasiaI	Unknown
NCT03164135	HIV	CCR5	CD34+ HSCT	5	N/A	To verify the clinical feasibility for CCR5‐KO CD34+ HSCT for hematological malignances in patients with HIV	Unknown
NCT03747965	Mesothelin (+) multiple solid tumors	PD‐1	CAR‐T	10	1	To verify the clinical feasibility of PD‐1‐KO CAR‐T cells for mesothelin (+) solid tumors	Unknown
NCT03545815	Mesothelin (+) multiple solid tumors	PD‐1 and TCR	CAR‐T	10	1	To verify the clinical feasibility of PD‐1‐KO and TCR‐KO CAR‐T cells for mesothelin (+) solid tumors	Recruiting
NCT04426669	Solid tumor	CISH	Engineered T cells	20	1/2	To verify the clinical feasibility of CISH‐KO TIL cell for solid tumor	Recruiting
NCT04438083	r/r renal cell carcinoma	CD70	Engineered T cells (CTX130)	107	1	To verify the clinical feasibility of CTX130 for r/r renal cell carcinoma	Recruiting
NCT04417764	Advanced hepatocellular carcinoma	PD‐1	PD‐1 KO T cell	10	1	To verify the clinical feasibility of TACE combined with PD‐1 KO cell infusion for advanced hepatocellular carcinoma	Unknown
NCT03044743	Advanced EBV‐associated malignancies	PD‐1	PD‐1 KO EBV‐CTL	20	1/2	To verify the clinical feasibility of PD‐1‐KO CTL for progression stage EBV‐induced gastric carcinoma, nasopharyngeal carcinoma and lymphoma	Unknown
NCT03655678	*β*‐thalassemia	BCL11A	CD34+ HSPCs (CTX001)	45	2/3	To verify the clinical feasibility of CTX001 for *β*‐thalassemia	Active, not recruiting
NCT04925206	*β*‐thalassemia	BCL11A	CD34+ HSPCs (ET01)	8	1	To verify the clinical feasibility of ET01s for *β*‐thalassemia	Recruiting
NCT03745287	SCD	BCL11A	CD34+ HSPCs (CTX001)	45	2/3	To verify the clinical feasibility of CTX001 for SCD	Active, not recruiting
NCT05477563	SCD and TDT	BCL11A	CD34+ HSPCs (ET01)	12	3	To verify the clinical feasibility of CTX001 for SCD and TDT	Recruiting
NCT03728322	TDT	HbB	iHSCs	12	1	To verify the clinical feasibility of transplantation HbB‐KO iHSC with TDT mutations	Unknown
NCT05565248	Glucose metabolism disorders	Immune evasiveness	Pancreatic endoderm cells (VCTX211)	40	1/2	To verify the clinical feasibility of pancreatic endoderm cells for glucose metabolism disorders	Recruiting
NCT03872479	Leber congenital amaurosis 10	CEP290	AAV5 (EDIT‐101)	34	1/2	To verify the specificity and efficiency of CEP290 targeting AAV and treatment effect	Active, not recruiting
NCT04037566	Hematological malignancies	HPK1	XYF19 CAR‐T	40	1	To verify the clinical feasibility of XYF19 CAR‐T cells for r/r CD19 (+) leukemia or lymphoma	Recruiting
NCT04244656	Multiple myeloma	BCMA	Engineered T cells (CTX120)	80	1	To verify the clinical feasibility of CTX120 for r/r multiple myeloma	Recruiting
NCT03398967	r/r Hematological malignancies	CD19, CD20, CD22	CAR‐T	80	1/2	To verify the clinical feasibility of the universal CD19/CD22 and CD20/CD22 CAR‐T therapy in progression stage hematological malignancies.	Unknown
NCT04035434	r/r B‐cell malignancies	CD19	Engineered T cells (CTX110)	143	1	To verify the clinical feasibility of allogeneic engineered T cells (CTX110) therapy in progression stage hematological malignancies.	Recruiting
NCT03166878	r/r CD19+ leukemia and lymphoma	CD19	Engineered T cells (UCART019)	80	1/2	To verify the clinical feasibility of UCART019 for r/r CD19+ leukemia and lymphoma	Unknown
NCT05397184	r/r T‐cell ALL	Base edited	CAR‐T	10	1	To verify the clinical feasibility of base edited allogenic CAR‐T in T‐cell ALL	Recruiting

clinicaltrials.gov; search date: 2023/03/27

VLP, virus‐like particle; VEGFA, vascular endothelial growth factor A; CISH, cytokine‐induced SH2 protein; TIL, neoantigen‐specific tumor‐infiltrating lymphocytes; iHSCs, induced hematopoietic stem cells; HSCT, hematopoietic stem cell transplantation; CCR5, CC chemokine receptor 5; PD‐1, programmed cell death protein 1; BCMA, B‐cell maturation antigen; EBV, Epstein‐Barr virus; TACE, transcatheter arterial chemoembolization.

### 4.2 Disease Diagnosis

The CRISPR‐Cas9 system's exceptional specificity has generated significant interest among researchers in the field of disease diagnosis. The seed region of sgRNA is particularly noteworthy, as even a single‐base pair mismatch can significantly reduce cleavage efficiency or render the target sequence completely unrecognized. Due to the high specificity of the seed region, researchers have employed it extensively for detecting various nucleic acid sequences or fulfilling specific objectives.^[^ ^173^, ^174^ ^]^

#### 4.2.1 Zika Virus (ZIKV)

**Figure 9:**
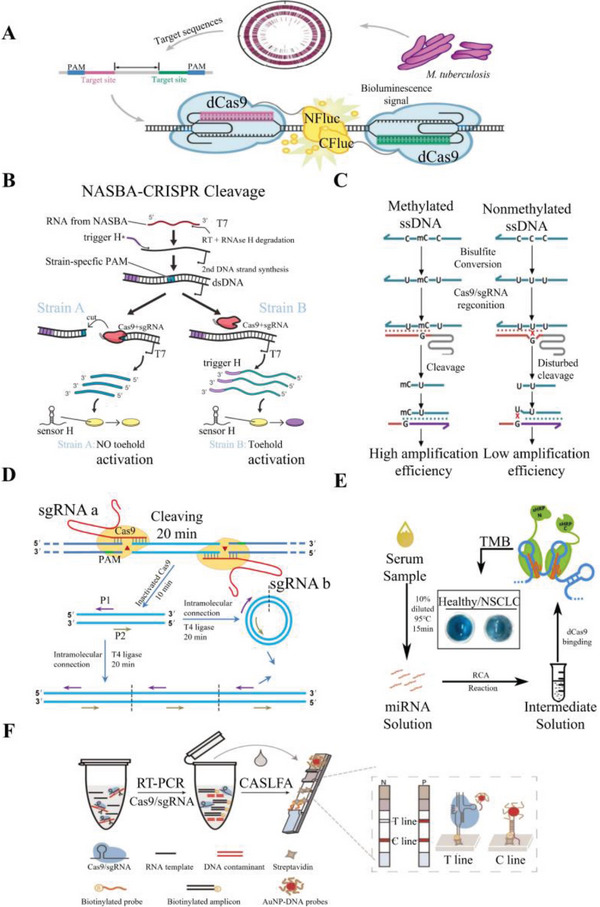
Schematic illustration of CRISPR‐Cas9 diagnosis technologies. Schematic illustration of A) dCas9‐based fluorescence reporter system. Reproduced with permission.^[^ ^176^ ^]^ Copyright 2017, American Chemical Society. B) NASBACC genotyping. Reproduced with permission.^[^ ^181^ ^]^ Copyright 2017, Elsevier Ltd. C) procedures for CAS‐EXPAR. Reproduced with permission.^[^ ^180^ ^]^ Copyright 2018, American Chemical Society. D) Procedures for CARP. Reproduced with permission.^[^ ^182^ ^]^ Copyright 2018, Springer Nature. E) Procedures for RCH detection of the miRNA. Reproduced with permission.^[^ ^179^ ^]^ Copyright 2018, American Chemical Society. F) Procedures for CASLFA. Reproduced with permission.^[^ ^32^ ^]^ Copyright 2021, John Wiley & Sons, Inc.

The first application of CRISPR‐Cas9 system in the diagnosis field was for the detection of ZIKV during its global spread in 2016. Pardee et al. employed an isothermal RNA amplification technology to duplicate pathogen sequences. They used toehold switch‐based RNA sensors to distinguish American ZIKV from African ZIKV and other similar diseases, such as dengue. The researchers designed a sgRNA that matched the U.S. ZIKV mutation region and showed a one‐base pair mismatch to the African ZIKV. The toehold switch‐based RNA sensor results showed that the CRISPR‐Cas9 system could only cleave U.S. ZIKV amplification products and distinguish them within 3 h, even at a relatively low virus concentration (**Figure** **9**B).^[^ ^31^ ^]^ This finding highlights the potential of the CRISPR‐Cas9 system in sequence detection and disease diagnosis to address global health crises.

#### 4.2.2 Human Papillomavirus

The limitations of the vaginal mucus barrier and universal primers have hindered accurate diagnosis of HPV, leading to false‐negative and false‐positive results for clinical doctors and patients. To address this challenge, a novel system combining CRISPR‐Cas9 and reverse polymerase chain reaction was constructed. This system involved three key steps: i) cleaving the HPV‐specific sequence using a pair of specifically designed sgRNAs, ii) ligating the cleaved DNA with DNA ligase, and iii) amplifying the ligated DNA with designed reverse primers. This system successfully detected nine subtypes of HPV, achieving a limit of detection (LoD) of 0.002 ng mL^−1^ (Figure 9D).^[^ ^175^ ^]^

#### 4.2.3 Tuberculosis (TB)

TB is a serious and challenging disease due to its elusive symptoms, difficulty in diagnosis, and complex treatment options. To address these challenges, Zhang et al. developed a novel molecular beacon‐based pair of dCas9 fluorescence probes capable of detecting low concentrations of TB. The probes were connected to a dCas9‐sgRNA pair with N‐firefly luciferase (NFluc) or C‐firefly luciferase (Cfluc). When the two Fluc‐dCas9 probes bound to the adjacent target site, luciferin was transferred to oxyluciferin by luciferase, resulting in the release of fluorescence. The TB sample demonstrated a 52‐fold stronger fluorescence intensity than the *Escherichia coli* sample, with an LoD of 5 × 10^−5^ nmol mL^−1^. These findings highlight the potential of the molecular beacon‐based dCas9 fluorescence probes to improve TB diagnosis and pave the way for more effective treatment options (Figure 9A).^[^ ^176^ ^]^

#### 4.2.4 MicroRNA

MiRNAs have been found to play multiple roles in various diseases, making them potential diagnostic markers. Among the most detectable miRNAs, circulating miRNAs have been widely applied in disease diagnosis, particularly in neoplastic diseases.^[^ ^177^, ^178^ ^]^ In a recent study, Qiu et al. employed the rolling circle amplification (RCA) technique to amplify Let‐7 family miRNAs from blood samples. They then mixed the amplification products with dCas9‐spilt‐HPR and sgRNA to change the color of 3,3′,5,5′‐tetramethylbenzidine for the identification of Let‐7a in patients with NSCLC. This miRNA detection system achieved 35.4 am LoD or the Let‐7 family and an fm level LoD for Let‐7a (Figure 9E).^[^ ^179^ ^]^

#### 4.2.5 Methylation Detection

Methylation detection is a complex and time‐consuming process that often requires specialized instruments and reagents. A new method utilizes the bisulfite conversion of unmethylated cytosine to uracil and the CRISPR‐Cas9 cleavage and nicking endonuclease system to cleave the target DNA fragment for use as primers in amplification. This innovative approach has demonstrated the ability to accurately distinguish single‐base‐level methylation in a convenient and low‐cost manner. These promising results have significant implications for the study of epigenetic modifications and their role in disease diagnosis and treatment (Figure 9C).^[^ ^180^ ^]^

#### 4.2.6 Anticontamination

PCR is a commonly used diagnostic tool for pathogen detection due to its affordability, rapidity, and sensitivity. However, the high sensitivity of PCR also makes it susceptible to false‐positive results caused by contamination. To address this issue, a contamination elimination system based on CRISPR‐Cas9 technology was developed. This system utilizes PAM‐implanted PCR to introduce a PAM sequence into the contaminating DNA, effectively eliminating it. The PAM‐implanted PCR approach significantly improves the anticontamination capacity of open‐tube PCR experiments and is a valuable tool for laboratories. This novel approach has the potential to enhance the accuracy and reliability of PCR‐based diagnostic tests, particularly in cases where contamination is a significant concern (Figure 9F).^[^ ^32^ ^]^

## 5 Challenges of the CRISPR‐Cas9 System in Translational Medicine

### 5.1 Off‐Target Effects

Off‐target effects are a significant concern and an unavoidable issue associated with gene editing tools. Studies have investigated the off‐target rates and the direct and long‐term side effects associated with these effects.^[^ ^183^ ^]^ For example, a quantitative EGFP disruption assay was performed to evaluated for single and double mismatch‐induced off‐target phenomena, by comparing the indel frequency of similar nucleic acid sequences. Interestingly, under specific conditions, the knockout efficiency of the mismatch site was higher than that of tightly matched sites. This finding raises important questions regarding the matching mechanism of the sgRNA and target sequence, which involves the PAM region and the seed region corresponding to the adjacent 3′ end of the PAM. Despite various complex mechanisms remaining unelucidated, the accuracy and efficiency of software prediction results are increasing with the support of artificial intelligence and a large amount of experimental data. Additionally, numerous free software options are available to address this issue, which is likely the best solution.^[^ ^184^ ^]^

### 5.2 Mitochondrial Genome Editing

Mitochondrial diseases are a group of oxidative phosphorylation disorders associated with mutant genes in either nuclear DNA encoding mitochondria or mitochondrial DNA (mtDNA).^[^ ^185^ ^]^ Although the functions and pathogenicity of some mitochondrial genes have been verified, the method for mitochondrial genome editing is still limited. The main difficulties lie in two areas. i) Editing efficiency. The nucleic acid and protein introduction mechanism for mitochondria remains unclear, and the delivery efficiency of Cas9‐sgRNA into mitochondria is low. ii) Repair mechanisms for mtDNA are yet to be elucidated. It is essential to overcome these challenges to develop effective treatments for mitochondrial diseases.

### 5.3 Delivery Vectors

The use of mainstream delivery vectors, such as lentivirus and AAV, presents several challenges, including potential immunogenicity, low loading capacity, insufficient targeting, limited barrier passing capacity, and restricted accessibility to solid tumors.^[^ ^186^ ^]^ Consequently, current in vivo CRISPR‐Cas9 therapies are limited to hematological disorders, and clinical CRISPR‐Cas9 therapies depend on endogenous cells, such as T cells and HSPCs. Therefore, the development of a safe, highly effective, and targeted vector is crucial for the broad applications of in vivo CRISPR‐Cas9 therapy.^[^ ^187^ ^]^ Research on CRISPR‐associated vectors, such as liposomes, lipid nanoparticles, and polymer nanoparticles, has progressed rapidly in recent years. We propose that these advanced nanomaterials can overcome the aforementioned challenges and facilitate the rapid development of CRISPR‐Cas9 therapy.

### 5.4 Ethical Issues

Gene editing technology has brought about significant advancements in the field of medicine, but it has also given rise to ethical concerns that need to be addressed. The current ethical issues mainly revolve around two points: embryo issues and equity issues. With regards to embryo issues, there is a consensus that embryo editing for reproductive purposes is prohibited, but other embryo‐related studies need to be conducted. These studies are critical for understanding the developmental process of embryos and related regulator factors. However, the sources of embryos are controversial, with some countries only permitting the use of embryos that cannot survive successfully, while others permit the use of healthy embryos as editing objects because they are more valuable for research. Equity issues are also of concern in gene editing technology. The research and development process of gene editing therapy is costly, and this may lead to wealthy individuals having the right to decide the direction of research and get priority access to treatment. In addition, it is crucial to establish stringent regulations for nonessential gene editing therapies designed for functional enhancement, as they have the potential to yield significant economic benefits. Therefore, despite the excellent progress in validating the effect of gene editing therapy, more complex and enduring ethical issues need to be addressed. It is important for researchers and policymakers to consider the ethical implications of gene editing technology and to ensure that it is used in a responsible and equitable manner.

## 6 Conclusion and Perspectives

Translational research on CRISPR‐Cas9 gene editing technology is rapidly advancing, with numerous clinical trials being conducted. Preclinically, CRISPR‐based tools such as knock‐in, knock‐out, activation, and interference have been developed and demonstrated to have a wide range of applications and powerful treatment effects in various diseases. Clinical trials of CRISPR‐Cas9 therapy have been conducted, with in vivo therapies successfully applied in cancers such as NSCLC and r/r ALL, as well as hereditary diseases like ATTR and SCD, showing promising results. Additionally, CRISPR‐Cas9 disease diagnosis tools are being applied in several fields. These studies provide confidence in the future clinical applications of CRISPR‐based therapies.

Although there have been remarkable advancements in the clinical application of CRISPR‐Cas9 gene editing therapy, there is still a significant gap in large‐scale implementation. A crucial aspect that requires attention is the systematic investigation of potential side effects of the CRISPR‐Cas9 system, which is currently lacking. While some studies have reported the elimination of off‐target editing after 18 months,^[^ ^45^ ^]^ investigations need to be extended to over ten years and conducted in a multidimensional mode, rather than just genome sequencing. Moreover, current clinical therapies are limited to hereditary diseases and nonsolid tumors, due to the limitations of traditional vectors like plasmids and viruses. Therefore, we propose the use of advanced nanomaterial vectors that could be optimized for in vivo CRISPR‐Cas9 therapy, and interdisciplinary research could lead to the next breakthrough. Recent developments in mitochondrial editing, such as the construction of mitochondrial targeting vectors and novel CRISPR‐free mitochondrial base editors, hold promise for further advancements in this field.^[^ ^188^, ^189^ ^]^

In summary, the CRISPR‐Cas9 system and its derivatives have made significant advancements in clinical gene editing therapy, highlighting its unique advantages. However, the long‐term safety implications of in vivo editing require further investigation, and a thorough demonstration of safety would greatly promote the CRISPR‐Cas9 system in future translational medicine. The development of mitochondrial targeting vectors and base editors also hold promise for further advancements in this field.

## Conflict of Interest

The authors declare no conflict of interest.

## Author Contributions

R.X.Z. and L.X.Z. contributed equally to this work and should be considered as co‐first authors. R.X.Z. and L.X.Z. analyzed the literature, prepared the figures and tables, and drafted the manuscript. R.P. and L.H.S. edited the manuscript. X.Y.H., F.F.Y., and K.Q.S conceived the study, supervised the work, and revised the manuscript. All authors have read and approved the final manuscript.
